# The Ion Channel Inverse Problem: Neuroinformatics Meets Biophysics

**DOI:** 10.1371/journal.pcbi.0020091

**Published:** 2006-08-25

**Authors:** Robert C Cannon, Giampaolo D'Alessandro

**Affiliations:** University College London, United Kingdom

## Abstract

Ion channels are the building blocks of the information processing capability of neurons: any realistic computational model of a neuron must include reliable and effective ion channel components. Sophisticated statistical and computational tools have been developed to study the ion channel structure–function relationship, but this work is rarely incorporated into the models used for single neurons or small networks. The disjunction is partly a matter of convention. Structure–function studies typically use a single Markov model for the whole channel whereas until recently whole-cell modeling software has focused on serial, independent, two-state subunits that can be represented by the Hodgkin–Huxley equations. More fundamentally, there is a difference in purpose that prevents models being easily reused. Biophysical models are typically developed to study one particular aspect of channel gating in detail, whereas neural modelers require broad coverage of the entire range of channel behavior that is often best achieved with approximate representations that omit structural features that cannot be adequately constrained. To bridge the gap so that more recent channel data can be used in neural models requires new computational infrastructure for bringing together diverse sources of data to arrive at best-fit models for whole-cell modeling. We review the current state of channel modeling and explore the developments needed for its conclusions to be integrated into whole-cell modeling.

## Introduction

Ion channels drive voltage-based signal processing within neurons and convert chemical signals into voltage changes at the synapses between cells. They can be distinguished by the ions that they allow to cross the membrane and by their response to chemical signals or changes in the membrane potential. More than 140 types of voltage-gated channels have been identified so far [1], with an even greater number of ligand-gated channels [2]. Some occur across many different cell types; others are specific to certain cell types or phases of neural development. As well as their role in neural signaling, ion channel activity is integral to a wide range of physiological processes, and abnormal channel behaviour is implicated in numerous pathologies including epilepsy, cystic fibrosis, and some forms of diabetes [3].

The development of patch-clamp techniques [4] has greatly improved access to functional properties of ion channels and has allowed the pharmacological and electrophysiological characterization of many channel types. Biophysical studies have elucidated the molecular transformations underlying the activity of certain channels, and the first full crystal structure was solved in 1998 [5] for a bacterial potassium channel. Although a variety of computational models have been used to assist in these analyses [6–10], very little of this information is used in integrative models of neurons where the behavior of a neuron or small network is studied with respect to the individual currents across the membrane either in the continuous limit [11] or stochastically [12]. Instead, many models of neurons still fall back on the pioneering work of Hodgkin and Huxley [13], which uses a different and more limited formalism from the one used by biophysicists [14]. Parameter values are often drawn from previous modeling studies, frequently with little choice but to use models derived for different preparations, at different temperatures, and even in different species [15,16], with the resulting need to adjust parameters in complex models to achieve observed behaviors [11,17].

The reason that ion channel research is so rarely used by neural modelers is that there is currently no mechanism to incorporate it in a whole-cell model. A model requires good coverage of the whole-channel dynamics in a relatively small parameter space; but channel studies often focus on details of particular aspects of behavior and may leave other areas relatively ill-defined. For example, the complex gating of T-type calcium channels has important functional implications [18], but the most detailed models focus on exploring key aspects of the gating in detail, such as inactivation [19] or selectivity [20], rather than on developing broad coverage of the whole behavior. The information provides a valuable constraint and can be used to test the validity of a model, but it cannot be used “as is” either to construct a new channel model or to modify an existing one because the data originally used to characterize the model are generally not available. In a few cases, e.g., [21], biophysical analysis has yielded complete models that reproduce macroscopic currents and that could be used “as is” in whole-cell models, but no voltage-gated channels have been characterized in such detail.

Improving on this situation requires two complementary developments: reliable storage, dissemination, and reuse of experimental data [22]; and computational tools to derive the best approximations to channel models from this data. This would allow models to be routinely refined and updated as new data became available, and should reveal where the models are least well-constrained as a guide to where experimental work is most needed. The computational side constitutes a classic inverse problem, albeit with extremely diverse source data. The forward process from the channel to the recording (including the recording setup, electrode properties, amplifier circuitry, etc.) can be well-characterized, but it does not have an explicit inverse to get back to the channel from the data. Instead, indirect methods must be employed.

The inverse approach differs from traditional data analysis and parameter estimation in two ways. First, its main objective is not to measure specific quantities believed to be part of the underlying system, but to deliver a computational equivalent with behavior that is as close as possible to that of the underlying system. Second, it avoids any form of model-specific analysis by comparing the model with data in the space of the raw data, with the result that the procedure can be scaled up to handle new preparations more readily. Naturally, if a model can be completely characterized, then the inverse approach ends up providing estimates for distinct physical properties of the underlying system, but in most cases the best approximation will be a more limited model that aggregates properties into a smaller set of parameters.

In this review we survey the mathematical methods and computational tools available for studying ion channels and map out the additional components needed to bridge the gap between what is available from studies of ion channels and what is needed to construct reliable models for use in computing neural activity. As increasing numbers of researchers turn to quantitative models of neurons to help refine and interpret their observations, it is vital that these models should be built from the best information available. Conversely, exploiting the wealth of ion channel studies to produce reliable channel models should facilitate new studies and make the characterization of neural activity dramatically more efficient. The next sections, respectively, introduce a) the channel-modeling problem, and discuss b) the potential for dedicated experimental work to facilitate model development, c) how diverse information from independent studies can be incorporated, and d) the greatest hurdles to be overcome in fully exploiting the wealth of empirical data that is collected.

## Deriving Channel Models as an Inverse Problem

With the growing availability of computational resources, numerical inverse approaches are increasingly used across a range of disciplines. Together with three dedicated journals, *Inverse Problems in Science and Engineering* (Taylor and Francis), *Journal of Inverse and Ill-Posed Problems* (VSP Publishing), and *Inverse Problems* (Institute of Physics), they address the question “what system gave rise to these observations?” usually by starting with a parameterized model that is expected to correspond to the real system for some point in its parameter space. A computational model of the recording process is built so that it can take any set of parameters and generate the data that they would have given rise to in exactly the same format as the experimental data. The model can then be compared to the real system in the space—that of the real data—where the most information is present. The forward calculation is then repeated over and over for different parameter sets guided by an optimization process to find the model or models that best represent the data.

Astronomers and geophysicists were among the first to exploit computational inverse methods, partly because, unlike other scientists, they have no option of interfering with the system under study, and partly because the forward processes giving rise to observational data can often be well-characterized. Even where direct inverses exist in the idealized case, such as deconvolving an image by the point spread function of the optics, it has long been recognized that better results can usually be obtained in practical problems by ignoring the direct inverse and using an iterative approach that incorporates other factors such as the power spectrum of the noise [23].

In its purest form, the approach specifically avoids any form of data processing, such as calculating an activation curve from voltage-clamp recordings of an ion channel, since these introduce unnecessary assumptions and reduce the dimension of the space in which models are compared. In effect, the motivation for this type of analysis is to reduce the dimension of the data so it can be handled more easily. But the inverse problem approach does not need this reduction and instead pushes the processing burden onto the computer.

In comparison with astronomical applications, the study of ion channels is characterized by relatively simple base models for the channels themselves, but by an enormous diversity of complex forward processes that give rise to distinct types of data. Historically, neuron models have tended to use the formulation of voltage-dependent ion channel gating first presented by Hodgkin and Huxley [13] in combination with conduction laws that are either purely Ohmic, or given by the Goldman–Hodgkin–Katz equations [24,25]. The Hodgkin–Huxley (HH) equations represent a channel as a set of serial independent gates: if all the gates are open, then the channel is open. Biophysicists, on the other hand, prefer [26] Markov models in which a channel is represented as a single entity that can be in any one of a small set of states with transitions between states governed by activation barrier–style equations (Box 1). Such models are also loosely known as “kinetic schemes,” particularly where the focus is on continuum behavior rather than on single-channel dynamics. This divergence between HH and Markov representations is more a matter of historical accident than a fundamental difference in approach. Colquhoun and Hawkes [27] discussed models in which a channel can be represented as multiple independent subunits each of which is described by a Markov scheme. With this extension, the HH models form the subset in which subschemes are restricted to having only two states. Until recently leading neural modeling software implemented only this subset with the result that investigators focused on these models. However, there is extensive evidence that gating complexes are often not independent: such models are unable to represent certain macroscopic behaviors [28] and studies of the proteins themselves show how the movement of one voltage sensor affects the probability of movement of the others [29]. Moreover, the ease with which Markov models can be extended to include new phenomena such as drug interactions [30] makes the Markov scheme a natural choice for channel models.

**Box 1. Mathematical Models of Ion Channels**

**Figure 1:**
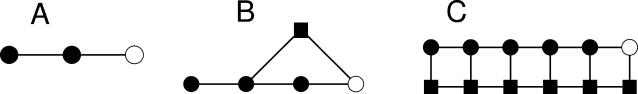
Examples of Markov Models (A) Three-state scheme considered in the text. (B) Best-fit model derived by Vandenberg and Bezanilla [60] for sodium currents in the squid giant axon using least-squares fitting to single-channel data. (C) T-type calcium model by Frazier et al. [61] reflecting structural constraints derived by exponential fitting of macroscopic currents. Open circles represent open (conducting) states. Filled circles and filled squares are closed and inactivated states, respectively. The distinction does not affect the structure or behavior of the model, but they are useful labels to tie the scheme to the phenomenology of channel behavior.

A Markov model ([31], Chapter 1) represents an ion channel as a collection of states and a set of transition probabilities between them: the key property of a Markov model is that the transition probabilities depend only on the states they connect and not on the previous history of the model. A simple case is shown in Figure 1A. The scheme can be written *C_1_ − C_2_ − O,* indicating three states, two closed (*C_1_* and *C_2_*) and one open *O*. A line between states indicates that a (stochastic) transition between them is allowed. In this case the allowed transitions are *C_1_ ↔ C_2_, C_2_ ↔ O*. The transition rates between states can be voltage- or concentration-dependent with appropriate temperature dependence as obtained, for example by using Eyring rate theory [32]. The most widely used expression for voltage-dependent forward, *f*, and backward, *b*, transition rates is

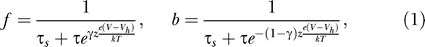
where *τ_s_* is the saturation transition time constant, *τ* is transition time constant, *k* is the Boltzmann constant, *T* the absolute temperature, *z* is the effective valence, *e* is the electron charge, *γ* is the gating asymmetry between the forward and backward transition rates, *V* is the membrane potential, and *V_h_* is the value of the potential at which the forward and backward transition rates are identical.

A Markov model can be solved either as a stochastic process or using a mean field approach. In the first case, it represents the state of a single channel: one assumes that the model is in a given state, e.g., *C_2_,* and that it either remains there or moves to another state according to an exponential probability distribution of rate *f* or *b*. In the second case a collection of identical channels is represented by a set of coupled differential equations for the fraction of the population in each of the states [27].

A Markov model of a channel can be designed starting from its molecular representation, with each state of the Markov model corresponding to a different configuration of the molecule, e.g., [26]. However, it is also possible to take a signal-processing approach to the design of Markov models: the required model is the minimal model that represents with sufficient accuracy the response of the channel to the stimulation protocols, e.g., [18]. The first approach arguably gives a better understanding of the structure and functioning of the channel and may ultimately be more appropriate when studying, for example, the genetic determination of channel behavior. The second approach leads to more economical numerical models that are more suitable for numerical simulations of large collections of channels and of neurons. We note that a channel may also be represented by a combination of independent Markov schemes such that the open fraction is the product of the open fractions for each component scheme [27]. This framework includes the HH model as a subset where each component scheme has only two states. Any compound scheme can also be represented as an equivalent single scheme, albeit a rather complicated one [32], but “multiplying out” the separate state combinations.

The inverse channel–fitting problem, i.e., how to derive the values of the parameters of a Markov channel model from measured electrophysiological currents, has been studied in detail for many years. First of all, equilibrium distributions of currents are insufficient to distinguish between channel models [8], and procedures have been developed to identify the equivalence classes of models with the same dwell times distributions [8,33]. It is therefore necessary to consider channel activity under dynamic conditions. Experimental data include macroscopic currents from a large collection of identical channels and single-channel currents. The latter provide noisy signals that are usually first converted to a best-approximation sequence of opening and closing transitions. Although an approach based on fitting of a two-dimensional distribution of dwell times has been suggested [34], most strategies consist of maximizing the likelihood of a model, defined as the probability that it gives rise to the observed sequence of open and shut times [6,7,27]. Over the years there have been refinements in this technique to take into account missed events due to insufficient time resolution of the experimental apparatus [35–37]. The use of macroscopic currents avoids problems of noise but presents its own statistical challenges. Recently techniques have been developed that use correlations in macroscopic current data to infer kinetic parameters [38,39]. There are freely available computer programs that implement these ideas, the “DC programs” [40,41] and QuB [42,43].

## Most Informative Data for Constraining Channel Models

The primary functional data for any model of an ion channel are in the form of the channel current in terms of the membrane potential and other environmental conditions as a function of time. The local conditions include the temperature, ionic concentrations on each side, and the concentrations of any ligands that act on the channel. The data may come from one or a few channels as in a cell-attached patch-clamp recording [4] or from a large number of identical channels expressed in a cultured cell line recorded in whole-cell mode. Each preparation and each recording method has its own uncertainties: are the expressed channels really representative of the wild type? Does the patch-clamp change the membrane properties and affect channel activity? The challenge of an effective inversion strategy is to incorporate all these uncertainties along with the corresponding data to arrive at the best available approximation to the underlying model.

A wide range of ingenious voltage protocols have been developed for studying ion channels, beginning with steps to different voltages and extending to ramps, spikes, and multiple pulses (e.g., [18,44]) The interest of these protocols is that they facilitate direct subsequent analysis such as the fitting of exponentials. At the other extreme, nonequilibrium response spectroscopy [45] applies rapidly fluctuating (up to 14 kHz) large-amplitude voltage signals. By careful choice of the dynamics of the signal, such as bandwidth and temporal asymmetry, the results can still be kept analytically tractable and provide information about properties of a channel that are not probed by stepped protocols. However, for an inverse approach, the feasibility of direct subsequent processing no longer matters and a much more extensive set of protocols can be used [39]. The most important factor is to maximize the discriminatory power of the data obtained. In the case of ion channels, this is also driven by the time limit on how long a channel can be held in its natural state before its activity changes due to washout of cellular constituents, other events in the cell, or general damage to the channel itself.

Once freed from any constraint to gather data that can be easily analyzed, one natural approach would be to subject channels to conditions that closely match those they normally experience. For example, a sequence of action potentials with a range of interspike intervals could be used for studying somatic channels in neurons. Alternatively, a sequence of steps to fixed potentials held for random time periods also exhibits a greater range of dynamics than conventional protocols. A good estimate of the discriminatory power for a given protocol can be readily obtained in simulations by measuring the sensitivity of the results to changes in the model parameters. One such case is shown in the last panel of Box 2, demonstrating how the more complex protocols provide tighter parameter constraints for a simple three-state model.

**Box 2. Parameter Constraints from Experimental Data**

The inverse approach proceeds by screening a range of possible Markov transition diagrams to find those diagrams and corresponding parameter sets that get closest to the experimental recordings. There are two main challenges in this approach: first, to determine how well a given dataset can constrain the most plausible model, and second, if the constraints are deemed adequate, to find that model using methods outlined in Box 1. The two are not independent since the strength of the constraint affects the performance of whatever fitting algorithm is used, and, fortunately, can be assessed without performing the full inversion.

**Figure 2:**
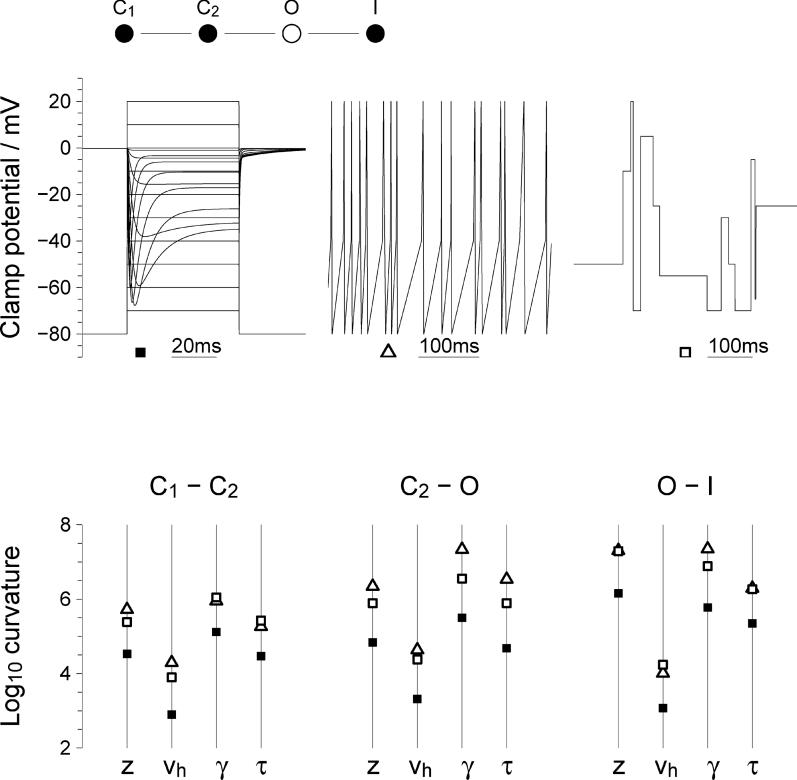
Parameter Constraints Arising from Different Command Profiles Sections of each command profile are shown in the first row. The strength of the constraints are shown in the second row for each of the twelve free parameters grouped into three voltage-dependent transitions. For each parameter, the symbols show the curvature of the error function around the exact model. Filled squares correspond to standard voltage steps, open triangles to a naturalistic spike-based waveform, and open squares to random steps.

Figure 2 shows three different voltage-clamp command waveforms, (top row). The first is a standard sequence of voltage steps separated by 900 ms at a holding potential of −80 mV (unpublished data). The model used is a linear four-state model approximating the behavior of a delayed rectifier sodium channel with parameters as given in Table 1. The current resulting from the first command sequence is shown overlaid.

**Table 1:**
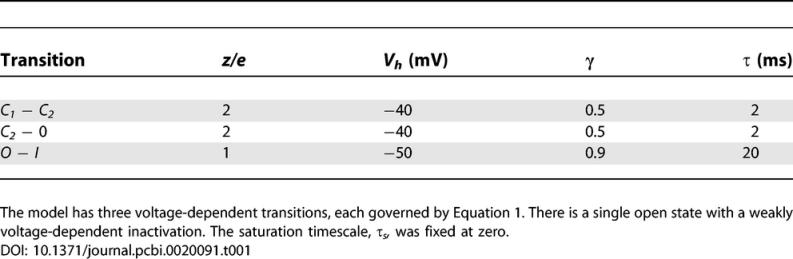
Parameters for the Example Model

The second command profile is a sequence of spikes with a Poisson-distributed interspike interval (mean 50 ms) and a steady ramp to −40 mV between spikes. The third profile has random jumps to potentials between −80 mV and +20 mV in multiples of 10 mV. Each value is held for a Poisson-distributed period, again with mean 50 ms. These are taken as canonical examples of the two styles and have not been adjusted in any way to fit this channel. All the command waveforms have a total duration of ten seconds.

The lower row shows the strength of the parameter constraints in the vicinity of the correct model for each of the twelve parameters (four parameters for each of three transitions) for the three different voltage commands indicated by filled squares, open triangles, and open squares, respectively. The quantity being displayed is the second derivative of the likelihood function with respect to the corresponding parameter. The scale is logarithmic so a difference of one unit indicates a constraint that is ten times stronger.

The two-complex waveforms provide constraints that are typically at least ten times tighter than the step sequence. This is not surprising since the step sequence has a long, quiet holding period occupying almost 90% of the stimulation. But it does demonstrate that the use of complex command waveforms that do not allow any direct analysis is not a problem for the method. More interestingly, some of the constraints, such as the one on the gating asymmetry *γ* for the opening transition, are almost another factor of ten stronger with spike stimulation than with the other protocols. Numerical experiments, such as adjusting the threshold in the spike profile or even just changing the seed for the random jump generator, suggest that sensitivity of other quantities also depends strongly on the details of the stimulation.

Perhaps the most intriguing aspect of this style of model construction is that the optimal protocol inevitably depends strongly on the channel being studied. Given the limited time available during an experiment, there are therefore significant advantages, in terms of confidence in the resulting model, in adjusting the protocol in real time in the light of the results. Given sufficient computing power, this could involve performing the full inversion during the experiment and iteratively refining the best-fit models as more data becomes available. Or it could involve a less computationally costly decision process as the experiment proceeds to determine the regions of most active dynamics and to concentrate on them.

Another significant feature of the approach is that the feasibility of the inversion varies nonlinearly with the volume of data: at low volumes of data, inversion is highly degenerate with no clear optimal model. But increasing the breadth of data can resolve the degeneracy, turning an underspecified problem into one which is easily solved. For example, models that cannot be distinguished under equilibrium conditions [8] can be resolved with nonstationary stimuli as long as one or more of the rates is stimulus-dependent. A further consequence is that models based on data from a range of sources and preparations can be expected to outperform models based on a single series of experiments or the work of a single laboratory, providing a strong incentive for investigators to share experimental data.

## Computational Methods

The examples in Box 2 illustrate that judicious choice of voltage-clamp command profiles can dramatically affect key quantities in the inversion algorithm that in turn will have a large effect on the feasibility of the fitting process. This is important because of the time-limited nature of voltage-clamp experiments and because of the presence of noise in any practical inversion. Short, well-constrained runs can be computed more quickly than longer, less well-constrained ones.

Although in the example the spiking command produces the tightest constraints, it is not necessarily the most appropriate command to use. The choice of command waveform should itself be treated as an optimization problem balancing a range of factors including: a) computational cost—channel models are easier to integrate if the stimulation has a step profile; b) screening efficiency—commands that can rapidly reject bad models could be very different from those that can refine good ones; c) off-minimum convergence—the example focuses on the final approach to a minimum, but it is equally important to help the fitting algorithm converge from more distant models.

These considerations demonstrate that before it is worth attempting the inverse problem on real channels, a simulation study should be used to work out what data should be collected to make the inversion feasible. Such a study can also indicate the level of confidence likely to be achievable in the resulting models.

In practical terms, a natural starting point for the search process is the set of previously published schemes and parameter sets for channels similar to the one under study as well as the previous results of the inverse process itself. The careful choice of this set is a one-off task with a significant effect on the success and speed of the inverse process. Occasional tests against a sequence of systematically generated possible schemes could be used to adjust the set of models for initial screening. Computational performance is heavily influenced by the type of optimization methods that can be applied. Gradients on the likelihood functions are easily computed and can be used in the final approach to optimal models, but local minima prevent the exclusive use of downhill methods. As Box 2 illustrates, the problem has an important degree of freedom in the choice of stimulation protocol. The example shows that this can be chosen so as to sharpen the minimum for particular parameters. A further possibility that is yet to be demonstrated is that other command sequences might improve convergence for models farther away from the optimum by smoothing the error surface or removing local minima.

The final test of a model is how well it can replace the real channel in its contribution to the activity of a neuron. Fitting to spiking protocols ensures the model sees the full dynamic range of the natural environment, but whole-cell models are also influenced by numerous factors that are still unquantified such as channel densities, localization, and even possible cooperation between channels [28]. These uncertainties are often addressed by fixing the channel models and fitting their densities [46]. The complexity of the system makes it unfeasible to include whole-cell behavior directly in the search for the best channel models, but it could be used where initial fitting produces distinct but equally plausible models.

## Use of Heterogeneous Data

The examples in Box 2 focused on current recordings in voltage-clamp mode that are collected specifically for the modeling task. But the majority of studies will have other primary objectives, so the results must be taken as is and accommodated within the general inversion process. This typically involves adding further free parameters that can soak up any systematic differences so that a close fit can still be achieved in much the same way as a blind deconvolution [47] separates a noisy signal into a best estimate for the original signal and an initially unknown point spread function.

There are also many other sources of data about the structure and kinetics of ion channels. A few channels have been crystallized and their three-dimensional structure is known [49]; many channels have been sequenced, giving indications about the number and ranges of groups involved in conformational changes. Where such data is not available for a particular channel in a given species, it may be available for channels in closely related species. These, and many other sources, provide information about the underlying mechanics of a channel. As with any form of statistical estimation, the challenge is first to estimate the value that can be derived from using a certain information source in the inverse problem, and second how to include it in the inversion process. Key questions for assessing the value are how strong a constraint the data provide; whether it influences parameters that are otherwise unconstrained; or whether the same constraints are more easily obtained elsewhere. These issues should all be addressed with respect to the final goal of the model. For example, from a biophysical perspective, the knowledge that four gating particles must move before the channel opens clearly determines that the scheme should have four closed states. From a neuroinformatics perspective, it is likely that a model that aggregates some of these states into a single state with a modified escape rate could achieve better overall tolerances.

Once these questions are satisfactorily answered, the technical process of incorporating the data is usually straightforward: an extra term is added to the error function with a weighting reflecting the combination of possible errors and uncertainties. For a biophysical model, the example above with four voltage-sensing groups translates directly to a prior, saying that schemes with other than four closed states are extremely unlikely. For a cell-level model, where the object is to derive a “semi–black-box” system that provides the best performance when computing whole-cell behavior, the prior will be much more complicated. It should take account of the tradeoff between an extensive state diagram and very uncertain parameters, or a small-state diagram and well-constrained parameters. The value of such models in neuroinformatics derives from their connection of two levels of description. They package up knowledge about protein conformation and dynamics in a form that can be used in studying whole cells. This is just one of many such connections needed in whole-cell modeling. It will be equally important to produce models of how cells use nuclear processes such as post-translation modification and channel transport and insertion to regulate which channels are actually present in the membrane.

Although many statistical methods exist to help address the question of how to choose the right level of complexity of the model to fit under these questions [49–51], their application to highly nonlinear systems such as ion channels still rests heavily on the experience and judgment of the investigator. As such, for practical applications it is often better to work directly with the ideas on which the theoretical analyses are based. The core observation is simply that extending a model generally makes it better able to fit the test data, but at some stage it begins fitting the noise, too, and this makes it less representative of other real data. The solution then is to ensure that there are always other data available which have not been used in deriving the model and that can therefore provide an independent test of its performance. An essential test is to measure the currents as the real channel is exposed to a potential sequence recorded from the cell type under study. A comparison with what the model does under the same conditions yields tolerances on the model and can flag any domains in which it is likely to fail.

## Access to Source Data

When treated as an inverse problem, the construction of channel models is most productive when there is a wide variety of different raw data available to be fitted. Ideally this should include recordings with complex waveforms in a range of different temperatures and chemical environments for the channel. For recordings to be of use in the fitting process they require extensive metadata detailing the preparation and recording conditions. The publication of such data and metadata is fully in line with emerging policies on data sharing [22,52] and the growing tendency of journals to accept extensive data supplements in their electronic versions.

In practice, however, it is difficult to provide and validate metadata for which there is no market: why take the time and trouble to package up data when there is no apparent further use for it? And how can one be sure the metadata is sufficient for the data to be useful, when the application that may eventually use it does not currently exist? Some of these concerns may be overcome by negotiated pairwise collaborations [53], but the most scalable solution is to develop the computational infrastructure first. Once a working software system is available, it creates a market for its own input data. It becomes worthwhile to prepare material to the point that it can contribute to modeling process because there is a clear outcome in improving the tolerances of fitted models. This mechanism can be seen in action for whole-cell models with the growth of databases such as ModelDB [54] and ChannelDB [55] around the modeling systems Neuron [11] and Genesis [56] and XPP [57]. The process has worked slightly differently in systems biology where there were already many software systems and it was the development of a single community-wide model specification, SBML [58], that facilitated the development of the BioModels database [59].

It should be stressed here that the model evaluation and optimization algorithms will form only a small part of any practical system for large-scale channel modeling. Much of the work will concern essential processes related to data and metadata management, data formats, data provenance, and user interaction with the system. From this perspective, the output models are not so much research products themselves, as the transient outputs of two more fundamental research efforts: first, the experimental recording themselves, and second, the software systems and additional input data (weights, priors, algorithm choices) that implement solutions to the inverse problem. Naturally, it is sure to be useful to fix and archive particular models, but this should not preclude their revision when more data is available or when new algorithms come online. Shifting the focus from building databases of model parameters to databases of original recordings with best-fit models as a transient product should also overcome objections that databases can become filled with unreliable information. Rather than offering a single set of parameters, it allows the user to pick the model most suited to their application, and to see how it was derived and how well it reproduces the original data.

Once sufficient data is available for largely automated harvesting and processing, the computational procedures outlined here can be seen in the role of an ongoing compression process. The resulting models provide a condensed version of the raw data: an investigator could use the model to find out what a particular channel is expected to do in any given circumstance without having to refer back to the original experiments. This type of interaction is increasingly important with the growth of multidisciplinary studies where the user of the channel model can benefit from incorporating the latest data but is unlikely to have the relevant expertise to work directly with the data. It is the software equivalent of routine developments in hardware where a new machine suddenly makes widely accessible a set of processes that used to require very specific training and expertise. And just as with hardware, although functional prototypes can be built that can be successfully operated by their creators, for the technology to achieve its full potential and gain widespread use, substantial investment is required in productizing the ideas and algorithms. The bioinformatics community has led the way in doing this in their own domain. Perhaps the ion channel inverse problem can be the first instance of this philosophy spreading across the boundary into neuroinformatics.

## Abbreviations

HH: Hodgkin–Huxley
